# Local-to-remote cortical connectivity in early- and adulthood-onset schizophrenia

**DOI:** 10.1038/tp.2015.59

**Published:** 2015-05-12

**Authors:** L Jiang, Y Xu, X-T Zhu, Z Yang, H-J Li, X-N Zuo

**Affiliations:** 1 Key Laboratory of Behavioral Science and Magnetic Resonance Imaging Research Center, Institute of Psychology, Chinese Academy of Sciences, Beijing, China; 2 Department of Psychiatry, First Hospital of Shanxi Medical University, Taiyuan, China; 3 University of Chinese Academy of Sciences, Beijing, China; 4 Faculty of Psychology, Southwest University, Beibei, China

## Abstract

Schizophrenia is increasingly thought of as a brain network or connectome disorder and is associated with neurodevelopmental processes. Previous studies have suggested the important role of anatomical distance in developing a connectome with optimized performance regarding both the cost and efficiency of information processing. Distance-related disturbances during development have not been investigated in schizophrenia. To test the distance-related miswiring profiles of connectomes in schizophrenia, we acquired resting-state images from 20 adulthood-onset (AOS) and 26 early-onset schizophrenia (EOS) patients, as well as age-matched healthy controls. All patients were drug naive and had experienced their first psychotic episode. A novel threshold-free surface-based analytic framework was developed to examine local-to-remote functional connectivity profiles in both AOS and EOS patients. We observed consistent increases of local connectivity across both EOS and AOS patients in the right superior frontal gyrus, where the connectivity strength was correlated with a positive syndrome score in AOS patients. In contrast, EOS but not AOS patients exhibited reduced local connectivity within the right postcentral gyrus and the left middle occipital cortex. These regions' remote connectivity with their interhemispheric areas and brain network hubs was altered. Diagnosis–age interactions were detectable for both local and remote connectivity profiles. The functional covariance between local and remote homotopic connectivity was present in typically developing controls, but was absent in EOS patients. These findings suggest that a distance-dependent miswiring pattern may be one of the key neurodevelopmental features of the abnormal connectome organization in schizophrenia.

## Introduction

Schizophrenia is a severe psychiatric disease associated with a series of mental impairments including hallucinations, delusions, loss of initiative and cognitive dysfunction. The widely distributed clinical symptoms have been increasingly hypothesized to be attributed to the abnormal brain networks in schizophrenia. This dysconnectivity hypothesis has been demonstrated by recent brain imaging studies,^1, 2, 3, 4^ which greatly advanced the elucidation of the pathological mechanism of schizophrenia. Another reemerged hypothesis is that the connectivity disruptions may reflect miswiring processes during brain development.^5, 6, 7, 8^

Early-onset schizophrenia (EOS) is a rare form of schizophrenia and provides a unique opportunity to directly examine the alterations in developing brains.^9, 10^ Previous studies on brain structure have demonstrated consistent findings regarding the disruptions of cortical morphology^10, 11, 12, 13^ and white matter microstructure,^14, 15, 16, 17^ implying altered brain structural connectomes in EOS. Abnormal growth profiles of the structural features have also been depicted recently and implied that this psychiatric condition may be associated with the abnormal maturational trajectories.^18, 19, 20^ The anatomical distance has been identified as a key factor in developing a brain connectome with optimized performance regarding both the cost and efficiency of information processing, and short-range connections are selectively matured into long-range connections during the normal brain development.^21, 22, 23^ Although distance-related disturbances of functional connectivity have been reported in schizophrenia across different age groups,^24, 25^ most resting-state functional magnetic resonance imaging (rfMRI)-based findings in schizophrenia have presented large discrepancies,^26, 27, 28^ which may partly reflect the grand challenges of human brain functional connectomics at the current stage.

Structural connectomics commonly uses diffusion MRI for mapping brain connectivity at a macroscale spatial resolution.^29, 30, 31^ Functional connectivity with rfMRI is another connectomics tool at a higher spatial resolution (millimeters) to human brain mapping (that is, functional connectomics) with its unique functional implications compared with structural connectivity. Functional connectomics has been widely employed to examine abnormal brain connectivity under various disease conditions.^32^ Specifically, an rfMRI-based brain graph can be constructed by thresholding a node-by-node correlation matrix of the time series. On the basis of this whole-brain graph, network connectivity metrics (for example, local efficiency, centrality, hubness, global integration and modularity) can be computed for further comparison across groups.^33, 34, 35^ This explicit graph reconstruction is highly dependent on the threshold selection, which possibly biases the estimation or introduces additional intra- and inter-individual variability of functional connectivity and leads to only moderate reliability of these metrics.^36, 37^

From the perspective of information transferring, the remote functional connectivity was based on the local functional connectivity and its neurodevelopment. Therefore, it is important for identification of the miswired connectivity profile in schizophrenia to investigate how the alteration of local connectivity leads to abnormal changes of remote connectivity. Regarding the previous findings that neurodevelopment of connectivity featured a short-to-long distance pattern, we propose a distance-related miswiring hypothesis on developing connectomes at macroscale. It expects that the local connectivity is altered in both EOS and adulthood-onset schizophrenia (AOS) patients, but during the developmental process of EOS, these alterations can lead further to abnormal remote connectivity in EOS and not in AOS. To test this hypothesis, we studied a sample of 88 rfMRI data sets from drug-naive and first-episode patients with EOS and AOS. We developed a novel threshold-free and surface-based approach to examine both local and remote functional connectivity. By employing 2dReHo, a highly reliable and neurobiologically meaningful metric of local functional homogeneity,^38, 39^ we first detected the locations or vertices showing abnormal local connectivity on the cortical mantel in the patients. In subsequent functional connectivity analysis, these locations with altered local functional homogeneity were employed as seed regions to further examine the disrupted remote or long-range functional connectivity in schizophrenia. This integrated computational framework not only avoids the explicit construction of a functional brain graph but also allows us to directly examine the vertex-wise local-to-remote connectivity profiles and test the distance-related miswiring hypothesis on developing connectomes in schizophrenia.^40^

## Materials and methods

### Participants

The sample consisted of 126 subjects, including 41 EOS patients (age range 9–17.9 years, 18 males), 28 AOS patients (age range 19–51 years, 15 males), 35 typically developing controls (TDCs, age range 7.5–17.5 years, 17 males) and 22 healthy adult controls (HACs, age range 18–51 years, 10 males). The subject recruitment was community-based and completed at the First Hospital of Shanxi Medical University. Written informed consent was obtained from individual participants or their guardians (TDC and EOS) prior to data acquisition. The Ethical Committee for Medicine of the First Hospital of Shanxi Medical University approved this study.

The clinical diagnosis was determined by the consensus of two consultant psychiatrists according to a Chinese version of the modified structured clinical interview from the Fourth Edition of the Diagnostic and Statistical Manual of Mental Disorders (DSM-IV) for patient version. Patients with other axis-I or axis-II comorbidity disorders were excluded. All patients were experiencing their first schizophrenic episode and were drug naive during the neuroimaging scanning. Clinical syndromes were rated using the Positive and Negative Syndrome Scale (PANSS).^41^ These patients had no history of other major neuropsychiatric illnesses, head injury and alcohol or drug abuse. All healthy controls were interviewed with the structured clinical interview from the DSM-IV nonpatient version. TDCs received this interview together with their guardians. No subject had any history of significant prior medical diagnoses, substance abuse or neuropsychiatric disorders. Subjects who had a first-degree relative with a history of severe mental disorders were excluded.

### MRI data acquisition

We employed a Siemens Trio 3.0 Tesla scanner (Erlangen, Germany) at the Department of Radiology, Shanxi Provincial Hospital, China. All EOS patients and TDC completed a T1-weighted structural MRI scan with a magnetization-prepared rapidly acquired gradient echo sequence (repetition time (TR)=1900 ms; echo time (TE)=2.95 ms; inversion time (TI)=900 ms; flip angle (FA)=9° field of view (FOV)=225 × 240 mm^2^; matrix=240 × 256; slice thickness=1.2 mm, 160 sagittal slices, no gap) and an 8.8-min rfMRI scan using an echo-planar imaging sequence (TR=2500 ms; TE=30 ms; FA=90° FOV=240 mm; matrix=64 × 64; slice thickness=4 mm, 32 axial slices, no gap, 212 volumes). All AOS patients and HAC underwent T1-weighted structural imaging acquired using a three-dimensional fast low-angle shot sequence (TR=14 ms; TE=4.92 ms; FA=25° FOV=230 mm; matrix=256 × 256; slice thickness=1.5 mm, 120 sagittal slices, gap=0.1 mm) and an 8.3- min rfMRI scan obtained using an echo-planar imaging sequence (TR=2000 ms; TE=30 ms; FA=90° FOV=192 mm; matrix=64x64; slice thickness=5 mm, 33 axial slices, no gap and 248 volumes). All the participants received the same instruction. For the rfMRI scans, participants were instructed to close their eyes and remain awake while lying quietly.

### Data preprocessing

The Connectome Computation System (https://github.com/zuoxinian/CCS) developed by our laboratory^42^ processed all MRI data by integrating the three common neuroimaging toolboxes^43, 44, 45^ as well as in-house MATLAB scripts, and comprising both structural (*preStruc*) and functional (*preFunc*) processing pipelines.^38, 39^ Briefly, the *preStruc* steps included noise removal,^46^ intensity correction, brain extraction, tissue segmentation, white and pial surface generation and deformation estimation between the spherical mesh and a common spherical coordinate system. The *preFunc* steps included time series despiking, slice timing and motion correction, functional brain extraction, four-dimensional global mean intensity normalization, nuisance regression of white matter, cerebrospinal fluid and Friston-24 motion curves,^47^ temporal filtering (0.01–0.1 Hz), linear and quadratic trend removal, co-registration between individual functional and structural images using a boundary-based registration (BBR) algorithm^48^ and projection of individual preprocessed four-dimensional time series onto a standard cortical surface *fsaverage5*.^49^

### Quality control procedure

The Connectome Computation System develops its quality control pipeline (QCP: http://lfcd.psych.ac.cn/ccs/QC.html) for both structural (*qcpStruc*) and functional (*qcpFunc*) images. In total, there are 88 subjects (26 EOS, 20 AOS, 25 TDC, and 17 HAC) passed the QCP for subsequent analysis (see Table 1 for a full description of demographical and QC information). More details of EOS demographics are presented in our previous work.^50, 51^ There is no difference in age between EOS and TDC (*P*=0.84) as well as no difference in age between AOS and HAC (*P*=0.22). The *qcpStruc* pipeline produces various figures that allow for visual inspection of the quality of the following procedures: (1) noise and motion blurring of individual T1 images; (2) brain extraction or skull stripping; (3) brain tissue segmentation; and (4) reconstruction of pial and white surfaces. An image showing a high level of scanning noise and motion blur will be excluded from further analysis. One author (X-TZ) inspected all QCP figures from *qcpStruc-2/3/4* and manually corrected the images/surfaces if relevant procedures failed. The *qcpFunc* pipeline also computes various metrics for quantifying the quality of the following procedures:^39^ (1) head motion during rfMRI such as the maximum translational distance (maxTran), maximum rotational degree (maxRot) and mean frame-wise displacement (meanFD)^49^ and (2) the minimal cost of the BBR (mcBBR).^52^ Any rfMRI data sets matching one of the following criteria were excluded from subsequent analysis: maxTran>3 mm, maxRot>3° or meanFD>0.35 mm.

### Local short-range functional connectivity: 2dReHo

To characterize the local short-range functional connectivity, we developed a surface-based functional metric, namely 2dReHo. This metric has been documented in our previous publications,^38, 39^ so we will only give a brief introduction to this method. For a given vertex on the surface grid (*fsaverage5*), we identified its nearest neighbors and computed Kendall's coefficient of concordance of the rfMRI time series of the nearest neighbors, including the vertex itself, as the local short-range connectivity of this vertex. This computation procedure was repeated for every vertex on the cerebral surfaces of both hemispheres to produce vertex-wise local connectivity (that is, 2dReHo) maps. This rank-based metric has been demonstrated to be very robust against various types of noise^37^ and characterizes the complexity of information processing.^53^ All individual 2dReHo maps were spatially smoothed with a Gaussian kernel with 10-mm full width at half maximum (FWHM) on *fsaverage5*.

Group comparisons were implemented with a series of general linear models with the covariates (variables of interests) of age, sex and diagnosis as shown in (equation 1), where the diagnosis was either EOS versus TDC or AOS versus HAC. To explore the age by diagnosis interactions, we also included an interaction term in the statistical model. To examine the global-level changes of local connectivity, we first calculated the global mean 2dReHo values as the averaged 2dReHo across all vertices of *fsaverage5* for all individuals and tested the changes with (equation 1). Four nuisance (not relevant) variables including meanFD, minimal cost of the BBR, Jacobian values of white surface transformation and global mean 2dReHo were then regressed out from 2dReHo at the group level to assess region-specific changes of local connectivity by testing the changes at each vertex of *fsaverage5*. The vertex-wise significance values for each contrast of group comparisons were corrected using a cluster-wise method based on the random field theory (cluster-defining *P*=0.01, cluster-level-corrected *P*=0.05).


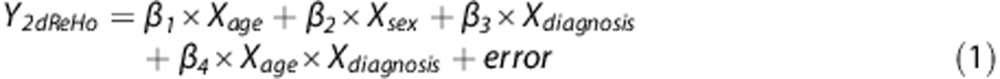


### Remote long-range functional connectivity: seed-based correlation

Limited by the nature of its methodology, 2dReHo can only characterize the impairments of local short-range functional connectivity in schizophrenic brains. To address this issue, we further developed a local-to-remote approach by combining the 2dReHo and seed-based functional connectivity (SFC) methods. Each cluster showing diagnosis effects on 2dReHo (group difference and diagnosis–age interaction) was used as a seed region for subsequent functional connectivity analyses to investigate how abnormal local connectivity will lead to remote connectivity changes in patients (Supplementary Figure S1). On the basis of individual preprocessed rfMRI time series that were spatially smoothed (FWHM=6 mm), the mean time series was computed across all vertices in the cluster and correlated with the time series of each vertex on *fsaverage5*. All individual correlation maps were converted into maps of Fisher *z*-values as individual SFC maps, which were further spatially smoothed (FWHM=10 mm) on the *fsaverage5* surface. Similar to 2dReHo, we first calculated global mean SFC values as the averaged SFC across all vertices of *fsaverage5* for all individuals and tested the global-level changes of remote connectivity with (equation 1). Three nuisance variables including meanFD, minimal cost of the BBR and global mean SFC^54, 55^ were then regressed out to assess region-specific changes of remote connectivity by testing the changes at each vertex of *fsaverage5*. The same correction approach as used in 2dReHo analyses was employed for multiple comparisons correction (cluster-defining *P*=0.01, cluster-level-corrected *P*=0.05/*N*), where *N* is the number of the surface clusters showing diagnosis effects on the local functional connectivity and is used to account for the additional statistical effects due to the multiple seeds employed).

## Results

### Global changes of cortical functional connectivity

**Figure 1:**
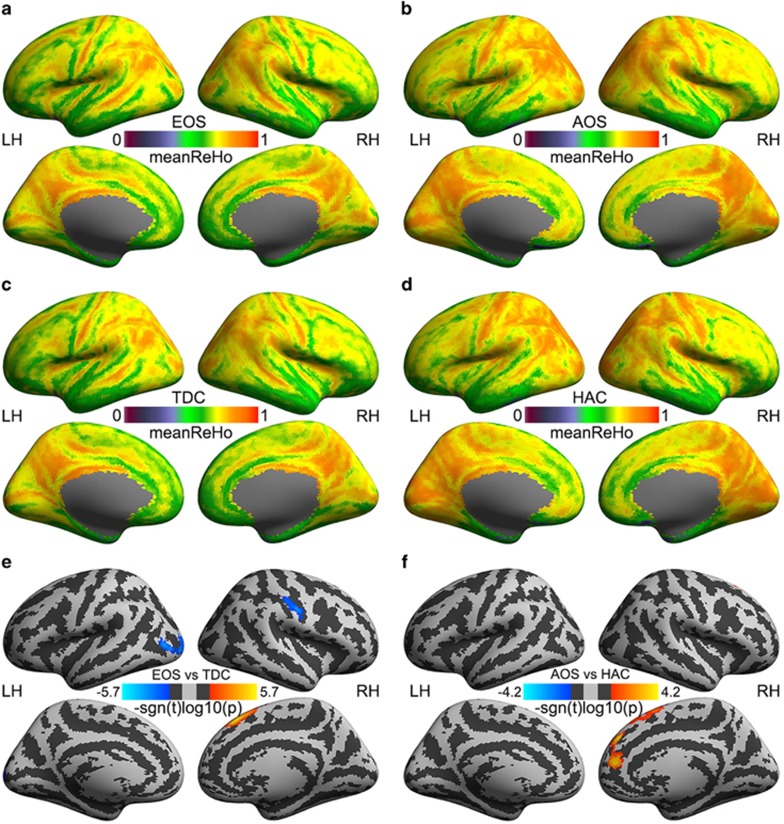
Mapping local functional homogeneity in schizophrenia patients and healthy controls. Group mean 2dReHo maps are depicted for early-onset schizophrenia (EOS; **a**), adulthood-onset schizophrenia (AOS; **b**), typically developing controls (TDCs; **c**) and healthy adult controls (HACs; **d**). Maps of group differences in 2dReHo are compared between EOS and TDC (**e**) as well as between AOS and HAC (**f**). The vertex-wise significance of group comparisons are measured with signed log10-transformed *P*-values and are rendered onto the cortical surfaces of the left hemisphere (LH) and the right hemisphere (RH). These inflated surfaces are defined by FreeSurfer as the *fsaverage5* surface model and visualized from lateral and medial views. Light-gray colors indicate the position of a cortical gyrus, whereas dark-gray colors show the position of a cortical sulcus.

**Figure 2:**
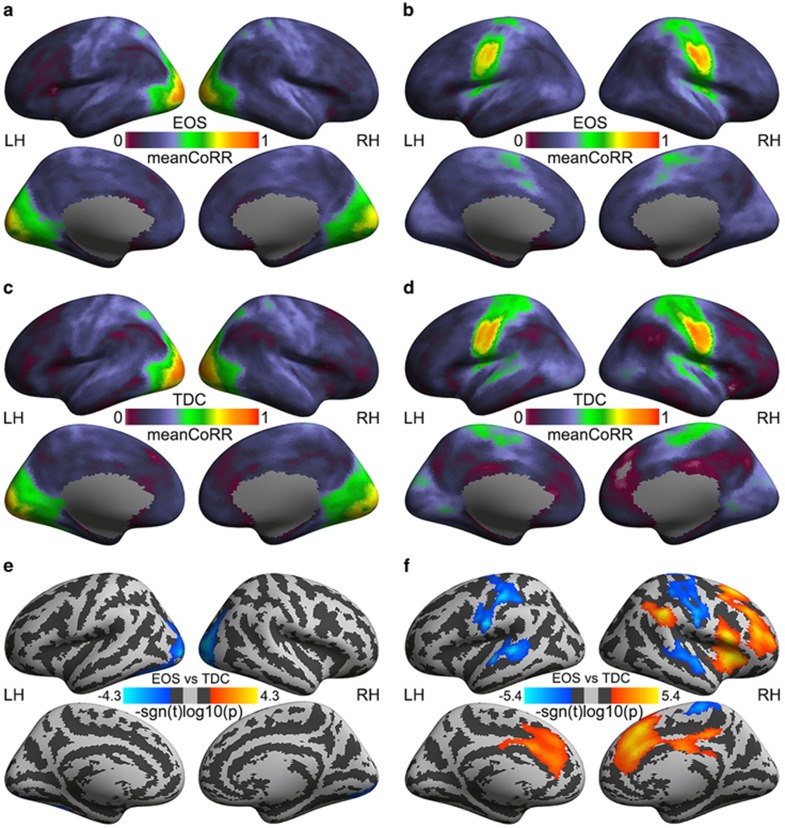
Mapping remote functional connectivity in early-onset schizophrenia (EOS) and typically developing controls (TDCs). Group mean correlation maps of two regions of interest as seeds (left column, left middle occipital cortex, MOC; right column, right postcentral gyrus, PosCG) are depicted for EOS patients (**a**, **b**) and TDC (**c**, **d**). Group differences in Fisher *z-*transformed correlations are compared between EOS and TDC (**e**, **f**). The vertex-wise significance of group comparisons is measured with signed log10-transformed *P*-values and is rendered onto the cortical surfaces of the left hemisphere (LH) and the right hemisphere (RH). These inflated surfaces are defined by FreeSurfer as the *fsaverage5* surface model and visualized from lateral and medial views. Light-gray colors indicate the position of a cortical gyrus, whereas dark-gray colors show the position of a cortical sulcus.

The group mean 2dReHo maps indicated that the whole-brain spatial patterns of the local functional homogeneity were highly similar across the four groups of subjects: EOS (Figure 1a), AOS (Figure 1b), TDC (Figure 1c) and HAC (Figure 1d). No significant difference in global mean values of local short-range connectivity (2dReHo) was observed. In contrast, region-specific changes of 2dReHo in patient–control diagnosis analyses were detected within five (three for EOS–TDC comparison and two for AOS–HAC comparison; see details of these clusters from Table 2) surface clusters, among which only two clusters exhibited significant group differences in their remote connectivity. The group mean maps of the SFC seeded by the two clusters are depicted in Figures 2a–d. No significant global changes of remote long-range connectivity were detectable for EOS versus TDC as well as for AOS versus HAC. No significant results for AOS–HAC comparisons of SFC and age–diagnosis interactions were detected. However, the individual differences in the global mean connectivity with these seeds can predict negative PANSS scores in the EOS patients.

### Region-specific changes of local functional connectivity

Beyond these large-scale patterns, significant changes were also detectable for vertex-wise local functional connectivity as revealed by the two-sample *t*-tests. We observed increasing 2dReHo of the right superior frontal gyrus (SFG) in both EOS and AOS patients (Figures 1e and f). AOS but not EOS patients exhibited significantly increased 2dReHo in the right medial prefrontal cortex (Figure 1f). In contrast, EOS patients demonstrated significant decreases of 2dReHo in the right postcentral gyrus (PosCG) and the left middle occipital cortex (MOC) compared with TDC (Figure 1e). Supplementary Figure S2 presents the reproducibility of the above changes of 2dReHo by using leave-one-out validation analyses, indicating the highest reproducibility of 2dReHo increases in the SFG, although it was much lower in the comparison of AOS patients versus HAC (Supplementary Figure S2B) than that in the comparison of EOS patients versus TDC (Supplementary Figure S2A). When comparing EOS patients with TDC, the decreases of 2dReHo in both the PosCG and MOC also demonstrated moderate-to-high reproducibility (Supplementary Figure S2A).

### Region-specific changes of remote functional connectivity

ReHo-inspired seed functional connectivity analyses detected widely distributed impairments of remote functional connectivity in EOS but not in AOS patients. Significant reductions of short-range connectivity in the left MOC were spatially extended to long-range homotopic functional connectivity between the left MOC and the right MOC (Figure 2e). The right PosCG showed a more spatially distributed pattern of long-range connectivity changes in EOS patients. Similarly, decreased homotopic functional connectivity was detected within the PosCG, which also exhibited reduced long-range connectivity with the bilateral superior temporal cortex. In contrast, this seed showed its strengthened functional connectivity with the right inferior precentral sulcus, inferior frontal gyrus, angular gyrus and insular and prefrontal cortex in EOS patients (Figure 2f).

### Functional covariance between local connectivity and remote connectivity

For each cluster showing significant changes of both local and remote functional connectivity in EOS or AOS patients, we examined the relationship between its local short-range connectivity and remote long-range connectivity by computing the Pearson's correlation coefficient between the average 2dReHo and the average Fisher *z-*value of the functional connectivity within the cluster. This across-subject covariance computation was performed for EOS, TDC, AOS and HAC. We found that the local connectivity of the left MOC exhibited a significant correlation with its homotopic connectivity in TDC (*r*=0.71, *P*<0.0001) but not in EOS patients. Similarly, we found the local connectivity of the right PosCG significantly correlated with its homotopic connectivity in TDC (*r*=0.56, *P*<0.004) but not in EOS patients. Fisher *z*-tests showed that the differences in the local–remote functional covariance relationship were significant between EOS patients and TDC.

### Diagnosis–age interactions on cortical connectivity

**Figure 3:**
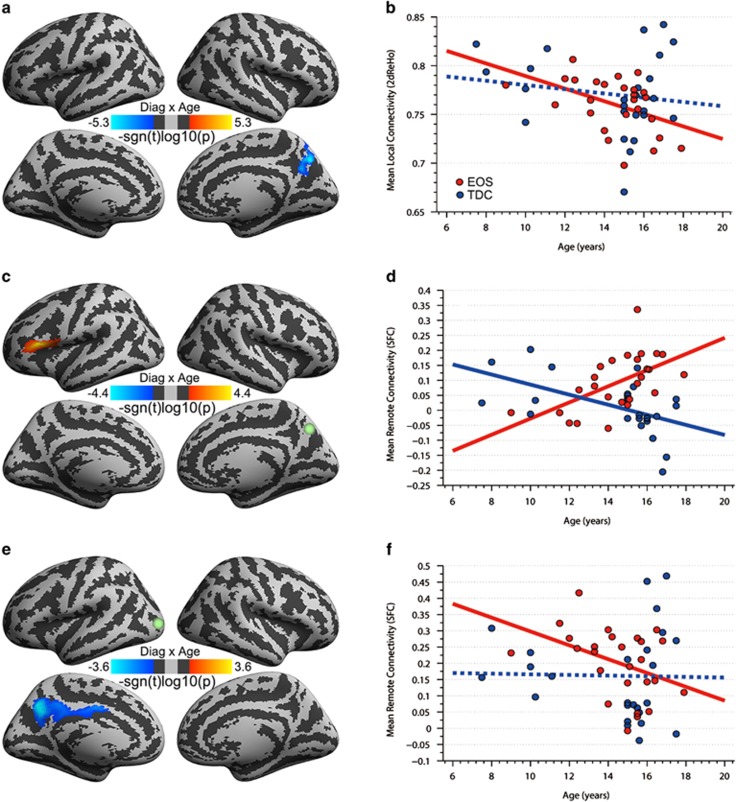
Mapping age–diagnosis (early-onset schizophrenia (EOS) versus typically developing controls (TDCs)) interactions on functional connectivity. The vertex-wise significance (the signed log10-transformed *P*-values) of interactions are visualized on the cortical surface for the local connectivity (2dReHo) (**a**) and the remote functional connectivity (correlation) seeded by the right precuneus (**c**: PCU) and the left middle occipital cortex (**e**: MOC), in which the light-green balls indicate the location of the two seeds. The details of these interactions are further plotted as scatter graphs in (**b**) 2dReHo, (**d**) PCU seed connectivity and (**f**) MOC seed connectivity. Each dot represents the individual connectivity metrics and each line indicates the connectivity correlation with age (red for EOS patients and blue for TDC; solid for significant and dashed for insignificant correlations).

Diagnosis–age interactions in local and remote functional connectivity were only detectable for EOS patient versus TDC comparisons (Figure 3). Regarding local connectivity, within the right precuneus (Figure 3a), EOS patients' 2dReHo decreased with age (Figure 3b, red line, *P*=0.021), whereas TDCs' 2dReHo did not have this relationship with age (Figure 3b, blue line, *P*=0.459). Meanwhile, the long-range connectivity between this region (Figure 3c) and the left inferior frontal cortex exhibited significant increases with age in EOS (Figure 3d, red line, *P*=0.002) but significantly deceased with age in TDC (Figure 3d, blue line, *P*=0.005). There was no detectable interaction effect on the remote functional connectivity of the right postcentral cluster. Interestingly, the left MOC exhibited SFC with the left precuneus significantly detectable for age–diagnosis interaction effects (Figure 3e) in EOS patients (Figure 3f, red line, *P*=0.044) and not in TDC (Figure 3e, blue line, *P*=0.921). All these findings survived cluster-level corrections for multiple comparisons.

### Correlation between functional connectivity and clinical symptoms

A significant positive correlation between the average 2dReHo within the right SFG cluster and a positive PANSS score was detected in AOS patients (*r*=0.47, *P*=0.049). No other significant correlations were observed for both EOS and AOS patients between the average 2dReHo or long-range functional connectivity within the clusters exhibiting significant group differences and the PANSS scores, including the positive, negative or general psychopathology scores, the susceptibility supplementary scale scores and the total scores.

## Discussion

We present new evidence regarding the profiles of distance-dependent miswiring of the connectome in EOS and AOS. Although local short-range functional connectivity demonstrated alterations in EOS and AOS, remote long-range connectivity within these local areas was mainly presented in EOS but not AOS. This distance-dependent miswiring pattern involved frontal, parietal, cingulate, temporal and occipital areas and significantly interacted with age in our developing sample. Considering the neurobiological indications of anatomical distance during human brain development, this study may shed new light on the disconnection and neurodevelopmental mechanisms underlying schizophrenia.

### Distance-dependent miswiring connectome pattern in schizophrenia

The first feature of the miswired connectome in schizophrenia is the impaired local short-range functional connectivity. Although many previous large-scale connectomics studies have shown the abnormalities of local network connectivity and modularity in schizophrenia,^26, 56, 57, 58, 59^ our findings present evidence on alterations of local connectivity at a very small spatial scale (~8 mm). At this resolution, large discrepancies existed in previous findings based on a regional homogeneity measure in three-dimensional volumetric space.^60, 61, 62, 63, 64^ Within these, two studies presented consistent findings with our results: (1) reduced ReHo in the left middle occipital gyrus^60^ and (2) increased ReHo in the right SFG.^60, 61^ All the inconsistencies may be attributed to the partial volume effect of the traditional ReHo method,^65^ causing difficulties in achieving biologically meaningful interpretations. The metric we developed avoided this shortcoming by measuring the local functional homogeneity of the cortical mantel and has been demonstrated to have very promising test-retest reliability^38^ and potential neurobiological significance related to functional hierarchy or complexity of information processing across both spatial^39^ and temporal^53^ domains. This allows us to interpret the reduction of local functional homogeneity at both the whole-brain level and specific regions as an indication of the reduced integration procedure associated with more complicated information processing in schizophrenia. In contrast, the enhancements of local functional homogeneity observed in the right medial frontal cortex might reflect an increased integration procedure of less complicated information, which was correlated with the cognitive deficits in schizophrenia patients.

Can this focal and localized functional dysconnectivity diffuse to remote long-range functional connectivity in schizophrenia? Our findings supported it. Two major types of long-distance functional connectivity were altered. The first was the interhemispheric connectivity between homotopic areas such as the MOC and PosCG. This finding has been reported many times.^66, 67, 68, 69^ An association between a negative PANSS score and the functional homotopic connectivity between the bilateral PosCG areas was observed in our recent study.^50^ In the present work, this connectivity demonstrated a similar connectivity–symptom relationship (*r*=−0.40, *P*=0.06). The second type of connectivity was remote functional connectivity between the PosCG as well as unimodal sensory areas and heteromodal association areas, which included both weakened connectivity in the sensory motor and superior temporal cortex,^51^ as well as strengthened connectivity in the dorsal lateral prefrontal cortex, insula and default network core hubs such as the PCC and angular gyrus. The presence of both decreases and increases of these across-modality functional connections likely indicates an imbalance in the functional integration between different functional modules in schizophrenic connectomes.

Recent advances on functional connectomics have revealed anatomical distance as one key factor in functioning the human brain.^70, 71^ Short-distance connections promote formation of functional segregation across network modules, whereas long-distance connections normally support functional integration between different modules through hub regions.^72^ The optimal balance between segregation and integration is fundamentally important for distributed brain networks to implement various mental and cognition processes.^73, 74, 75^ Previous connectomics studies on schizophrenia guided by the graph theory have reported disruptions of both network modularity and rich clubs,^26, 56, 76, 77, 78, 79^ implying a failure in preserving the optimized network architecture of information processing. Our findings demonstrated, for the first time, a miswiring connectome pattern associated with this failure in schizophrenia, which featured impaired local connectivity in both primary sensory and frontal modules, as well as subsequent alterations of remote connectivity between the unimodal primary (visual and sensory motor) modules and heteromodal association (frontal, parietal and temporal) modules.

### Neurodevelopmental underpinnings of the miswired connectomes

One advantage of the present work is the medication-free and first-episode sample of schizophrenia patients across both child-/adolescent-hood and adulthood. This offered us an opportunity to examine the miswired connectivity profiles during brain development and maturation without the confounding effects of medicine and disease duration.^80, 81^ Previous studies have proposed a ‘local-to-distributed' functional connectivity organization pattern during normal brain development,^82^ which would weaken local connectivity and strengthen remote connectivity. Our analyses (blue dashed lines in Figure 3) demonstrated a trend of this pattern in TDCs, although it was not statistically significant due to the limited sample size. We observed an altered version of this developing pattern in EOS (red lines in Figure 3). Specifically, the right precuneus, a final hub product of the human brain connectome during development process,^83, 84^ exhibited an over-weakened local functional connectivity profile with age compared with TDCs. This hub's homotopic region also showed weakened age-related remote functional connectivity with the left MOC in EOS patients. The functional connectivity between the right precuneus and lateral prefrontal cortex exhibited distinct patterns of age-related changes. TDC showed integration (positive) to segregation (negative) changes with age increases,^85^ implying a normal developing profile of connectivity between the two high-order association areas. The inversed pattern in EOS patients may indicate a misbalance between segregation and integration across functional modules. We speculated that these findings reflected the connectome miswiring process that exhibits an off of functional connectivity preference selection for anatomical distance during development. The perturbations to the ‘local-to-distributed' developmental process due to both genetic and environmental risks related to schizophrenia may partly underlie this bias in EOS. Of note, the consistent increases in local connectivity observed in both EOS and AOS patients may be less developmentally relevant and instead may be a shared disease outcome.^86^ In contrast, the inconsistent alterations in local connectivity between EOS and AOS may indicate developmental effects on functional connectivity in schizophrenia.

One possible underpinning of the miswired connectome during development may be related to the relationship between local connectivity and remote connectivity. We assessed functional covariance measured by the correlation between local functional connectivity and remote functional connectivity, and only detected the local–remote functional covariance for homotopic connectivity in TDCs, whereas EOS patients did not show such a relationship. Previous studies have demonstrated neurodevelopmental relevance of both structural and functional covariance,^39, 87, 88^ and we thus thought that this observation helped to understand the altered connectivity pattern of the unimodal sensory areas during development, considering that this altered functional integration with the heteromodal areas was likely associated with the disconnected homotopic regions.

### Methodological issues

Several issues must be considered when interpreting our findings. First, although our study included both EOS and AOS patients with a broader age range (9–51 years) compared with previous studies, the sample size was relatively moderate. A larger sample would significantly improve the statistical and prediction power of the findings, especially those with statistically significant trends. Second, the age-related changes of distance-based functional connectivity were derived from cross-sectional data sets and thus our postulation on developmental effects must be treated as exploratory. A longitudinal follow-up design would allow us to examine the developmental trajectories of both local and remote connectivity directly in both patients and healthy controls, although we would face challenges in controlling for the medication effects. Finally, 2dReHo was first employed to detect abnormal alterations in local connectivity, and then by taking the clusters with significant group differences in 2dReHo as seeds to detect altered remote long-distance connectivity. This local-to-remote connectivity methodological framework is purely driven by local connectivity and can reveal a specific distance-dependent miswiring pattern, which indicates how the abnormal connectivity at local scales lead to that of global scales. Of note, this approach precludes the finding of regions showing only long-range connection problems with the absence of local-connection alterations. There is a possibility of the bias in long-distance connectivity abnormality toward EOS, as AOS may show long-range connection problems within other regions with no local-connection deficits. Therefore, our findings may only reflect specific parts of a more general miswiring pattern in connectomes of patients. Other methods, such as a vertex-wise whole-brain functional connectivity analysis, would give more insights into long-distance connection alterations in the future, although relevant limitations need to be addressed first (see Introduction).

## Conclusion

Both localized and distributed information processing associated with functional connectivity is significantly altered in schizophrenia. This biased distance-dependent connectivity pattern emerged as a key feature of miswired connectomes throughout the human brain development in schizophrenia. Our findings provide a converging framework to examine the effects of genetic risk and environmental event factors on psychosis in the developing brain, demonstrating the necessity of delineating the normative trajectories of the human brain connectomes.^42, 89, 90, 91, 92^

## Figures and Tables

**Table 1:** Participant information

	*EOS (*N=*26) mean (s.d.)*	*TDC (*N=*25) mean (s.d.)*	*AOS (*N=*20) mean (s.d.)*	*HAC (*N=*17) mean (s.d.)*
Age, years	14.51 (1.94)	14.37 (2.97)	26.40 (8.01)	30.29 (11.01)
Gender (males)	13	12	9	8
meanFD^a^(mm)	0.15 (0.09)	0.20 (0.12)	0.15 (0.13)	0.14 (0.12)
gReHo^b^	0.66 (0.04)	0.69 (0.03)	0.71 (0.05)	0.71 (0.06)
gSFC (rMPFC)^c^	0.26 (0.15)	0.30 (0.14)	0.31 (0.12)	0.31 (0.17)
gSFC (lMOC)^d^	0.26 (0.15)	0.31 (0.13)		
gSFC (rPosCG)^e^	0.27 (0.14)	0.26 (0.14)		
gSFC (rPCU)^f^	0.26 (0.13)	0.26 (0.15)		
gSFC (rSFG)^g^			0.29 (0.14)	0.31 (0.17)
PANSS_positive	12.29 (4.31)		20.45 (4.02)	
PANSS_negative	12.83 (5.07)		15.45 (6.81)	
PANSS_general	28.71 (6.91)		39.90 (7.45)	
PANSS_suppl	4.04 (1.57)		5.80 (3.29)	
PANSS_total	57.88 (13.79)		81.60 (17.13)	

Abbreviations: AOS, adulthood-onset schizophrenia; EOS, early-onset schizophrenia; gReHo, global mean 2dReHo; gSFC, global mean seed-based functional connectivity; HAC, healthy adult control; lMOC, left middle occipital cortex; meanFD, mean frame-wise displacement; rMPFC, right medial prefrontal cortex; rPCU, right precuneus; rPosCG, right postcentral gyrus; rSFG, right superior frontal gyrus; TDC, typically developing control.

a meanFD is the mean frame-wise displacement for in-scanner head motion.

b gReHo is the global mean of 2dReHo across the whole cerebral cortex.

c gSFC (rMPFC) is the global mean connectivity of the right medial prefrontal cortex (rMPFC) with all vertices across the whole cerebral cortex.

d gSFC (lMOC) is the global mean connectivity of the left middle occipital cortex (lMOC) with all vertices across the whole cerebral cortex.

e gSFC (rPosCG) is the global mean connectivity of the right postcentral gyrus (rPosCG) with all vertices across the whole cerebral cortex.

f gSFC (rPCU) is the global mean connectivity of the right precuneus (rPCU) with all vertices across the whole cerebral cortex.

g gSFC (rSFG) is the global mean connectivity of the right superior frontal gyrus (rSFG) with all vertices across the whole cerebral cortex.

**Table 2:** Abnormal distance-dependent cortical connectivity in schizophrenia

*Region*	*Hemisphere*	*Cluster size (vertices)*	*Peak location (*X, Y, Z*)*^a^	*Significance* −*sgn(t) log*_*10*_*(*P*)*
*I) Local connectivity: EOS*^b^ *versus TDC*^c^				
Middle occipital cortex (MOC)	Left	120	(−36, −83, 2)	−2.925
Postcentral gyrus (PosCG)	Right	83	(45, −9, 26)	−1.928
Superior frontal gyrus (SFG)	Right	58	(11, 17, 57)	1.816
				
*II) Local connectivity: AOS*^d^ *versus HAC*^e^				
Medial prefrontal cortex	Right	60	(7, 54, 26)	1.772
Superior frontal gyrus	Right	59	(11, 17, 57)	1.649
				
*III) Remote connectivity with the left MOC: EOS versus TDC*				
Middle occipital cortex	Right	310	(21, −97, 9)	−4.133
				
*IV) Remote connectivity with the right PosCG: EOS versus TDC*				
Precentral gyrus	Left	449	(−52, −4, 36)	−4.895
Superior temporal cortex	Left	230	(−53, −38, −4)	−3.415
Medial prefrontal cortex	Left	225	(−8, 7, 34)	2.863
Postcentral gyrus	Right	603	(62, −8, 26)	−5.873
Superior temporal cortex	Right	192	(65, −16, 3)	−2.612
Prefrontal/cingulate cortex	Right	978	(39, 41, 20)	6.088
Inferior precentral sulcus/inferior frontal cortex/insula	Right	388	(37, 7, 22)	5.433
Angular cortex	Right	219	(56, −36, 28)	2.337

Abbreviations: AOS, adulthood-onset schizophrenia; EOS, early-onset schizophrenia; HAC, healthy adult control; TDC, typically developing control.

a Coordination in the standard space (Talairach).

b Early-onset schizophrenia.

c Typically developing controls.

d Adulthood-onset schizophrenia.

e Healthy adult controls.
