# Supplementary material for: Local-to-remote cortical connectivity in early- and adulthood-onset schizophrenia
Source: Transl Psychiatry. 2015 May 12;5(5):e566–. doi: 10.1038/tp.2015.59 (PMC4471290; doi:10.1038/tp.2015.59)
Supplement: Supplementary Information [file tp201559x1.pdf]

Supplementary Materials

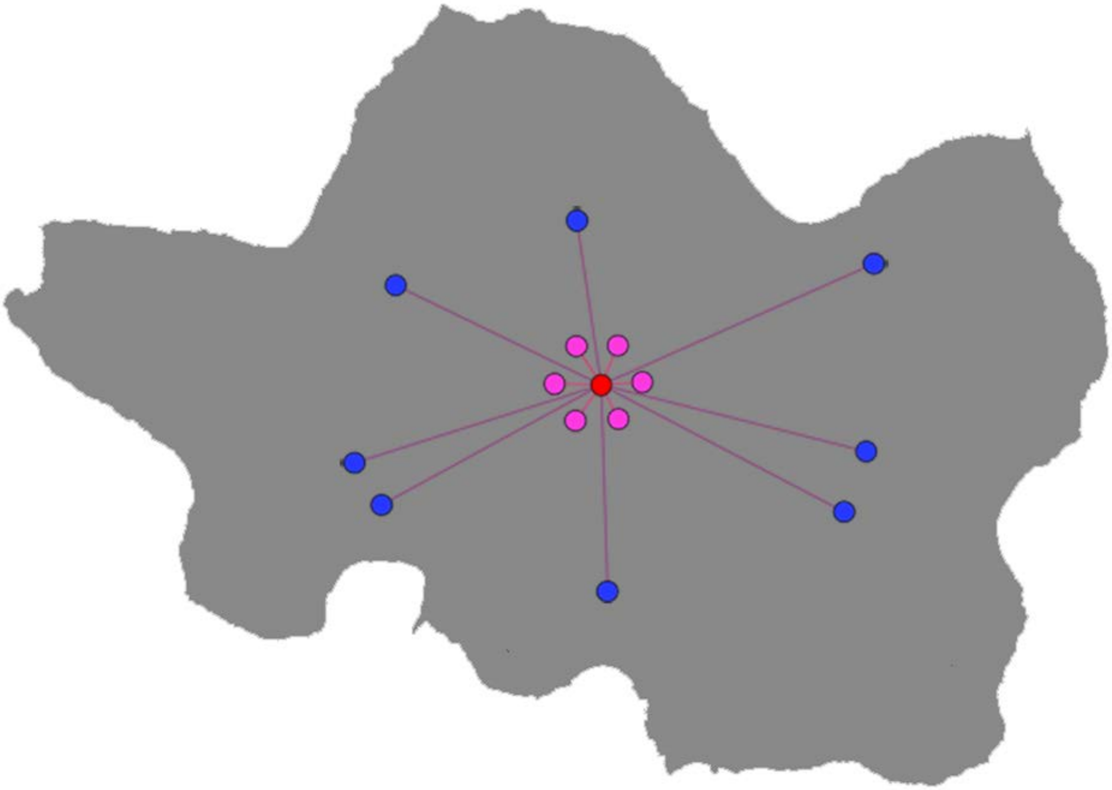

Figure S1. Sketch map of local and remote cortical functional connectivity. For a given vertex (red) on the surface grid of the human cerebral cortex (light gray), its local connectivity is the functional homogeneity of the time series from the vertex and its 6 nearest neighbors (pink). Meanwhile, its remote connectivity is measured by the temporal correlation between its time series and the times series of a remote vertex (blue).

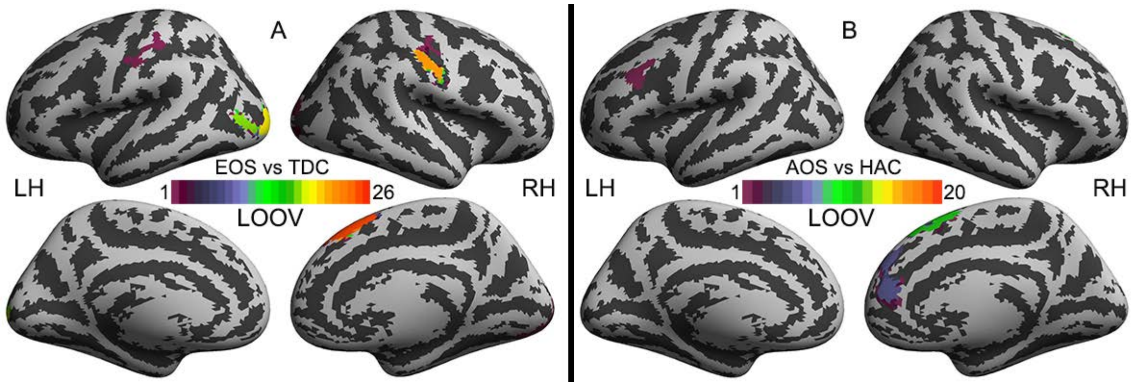

Figure S2. Leave-one-out validation (LOOV) on reproducibility of group differences in local functional homogeneity between schizophrenia patients and healthy controls. The vertex-wise numbers of replications of group comparisons (early onset schizophrenia versus typically developing controls: EOS vs. TDC; adulthood-onset schizophrenia versus healthy adult controls: AOS vs. HAC) are rendered onto the cortical surfaces of the left hemisphere (LH) and right hemisphere (RH). These inflated surfaces are defined by FreeSurfer as the *fsaverage5* surface model and visualized in lateral and medial views. Light gray colors indicate positions of a cortical gyrus whereas dark gray colors show the position of a cortical sulcus.
